# A Novel Process for Manufacturing High-Friction Rings with a Closely Defined Coefficient of Static Friction (Relative Standard Deviation 3.5%) for Application in Ship Engine Components

**DOI:** 10.3390/ma15020448

**Published:** 2022-01-07

**Authors:** Wojciech S. Gora, Jesper V. Carstensen, Krystian L. Wlodarczyk, Mads B. Laursen, Erica B. Hansen, Duncan P. Hand

**Affiliations:** 1 Institute of Photonics and Quantum Sciences, School of Engineering and Physical Sciences, Heriot-Watt University, Edinburgh EH14 4AS, UK; k.l.wlodarczyk@hw.ac.uk (K.L.W.); d.p.hand@hw.ac.uk (D.P.H.); 2 MAN Energy Solutions, 2450 Copenhagen, Denmark; jesperv.carstensen@man-es.com (J.V.C.); ericab.hansen@man-es.com (E.B.H.); 3 TRD Surfaces ApS, 2840 Holte, Denmark; mbl@trdsurfaces.dk

**Keywords:** laser surface texturing, surface functionalization, high friction

## Abstract

In recent years, there has been an increased uptake for surface functionalization through the means of laser surface processing. The constant evolution of low-cost, easily automatable, and highly repeatable nanosecond fibre lasers has significantly aided this. In this paper, we present a laser surface-texturing technique to manufacture a surface with a tailored high static friction coefficient for application within driveshafts of large marine engines. The requirement in this application is not only a high friction coefficient, but a friction coefficient kept within a narrow range. This is obtained by using nanosecond-pulsed fibre lasers to generate a hexagonal pattern of craters on the surface. To provide a suitable friction coefficient, after laser processing the surface was hardened using a chromium-based hardening process, so that the textured surface would embed into its counterpart when the normal force was applied in the engine application. Using the combination of the laser texturing and surface hardening, it is possible to tailor the surface properties to achieve a static friction coefficient of ≥0.7 with ~3–4% relative standard deviation. The laser-textured and hardened parts were installed in driveshafts for ship testing. After successfully performing in 1500 h of operation, it is planned to adopt the solution into production.

## 1. Introduction

Surface functionalisation has seen a significant uptake in industrial applications over recent years. Appropriate modification of surface topography can be achieved using various processes including, but not limited to, ion beam milling [1], coating [2], embossing [3], lithography [4], and laser surface texturing [5]. The flexibility of laser sources, their relative ease of automation, and their high precision mean that they are increasingly the mode of choice for surface modification, in particular with the relatively low-cost and highly robust fibre laser systems now commercially available. Such lasers are cost-effective and low maintenance, with suitable pulse parameters for surface modification. As a result, many research groups have focused their efforts on laser surface texturing to achieve different functional surfaces. This includes surface modification for aesthetic purposes [6,7], encryption of information [8], facilitating other processes (e.g., laser welding of dissimilar metals [9,10]), altering water-repellent properties [5,11,12], generating anti-icing [13] and anti-bacterial [14] surfaces, and modifying the tribological properties of surfaces, in particular reducing wear [15,16].

When focusing on the ability to tailor friction properties of surfaces, the majority of research has been focused on the reduction of the coefficient of static friction. This relies on the creation of various types of grooves and/or dimples. Such structures work either as lubricant reservoirs or as traps for any debris that might be created or introduced in the system [15,16,17,18,19]. The decrease in the friction coefficient is strongly dependent on the diameter, depth, and density of features created on the surface of the sample [20].

Even though laser surface modification for a low coefficient of friction is quite heavily researched, there is little work reported on increasing the coefficient of friction by means of laser surface texturing, despite some very promising results [21,22] where a set of craters or other features are created on the surface.

This paper focuses on an engineering requirement for surfaces with a high coefficient of static friction. Such surfaces are used in a marine engine driveshaft assembly to transfer power whilst protecting against damage. If the applied torque exceeds a specific level, the friction ring is designed to slip to prevent significant damage to the whole drivetrain. This double functionality requires the coefficient of static friction to be within a narrow range to ensure reliable operation. The current solution is to use a thermal spray coating to create a high-friction surface; however, this process results in the generation of non-uniform surface structures, in which the relative standard deviation of the coefficient of static friction exceeds 10% (typically between 13% and 14%). The requirement for this application is a minimum coefficient of static friction of 0.6. If the coefficient is too low, unintentional slippage can occur. On the other hand, if the coefficient is too high, excessive torque can be transferred to the drive train, leading to serious (catastrophic) damage to the component. Both situations give rise to significant extra costs, and application therefore calls for tighter control of the coefficient of friction. Such friction discs are single use, in that they are replaced if a slip occurs. To facilitate this, we have developed a highly repeatable two-step process that consists of: (i) laser surface structuring to provide a well-defined topography and (ii) subsequent deposition of a hard chromium carbide layer. In this article, we describe the development and characterization of this process and its application to manufacturing friction rings and discuss their performance during ship engine testing at sea.

## 2. Materials and Methods

**Figure 1:**
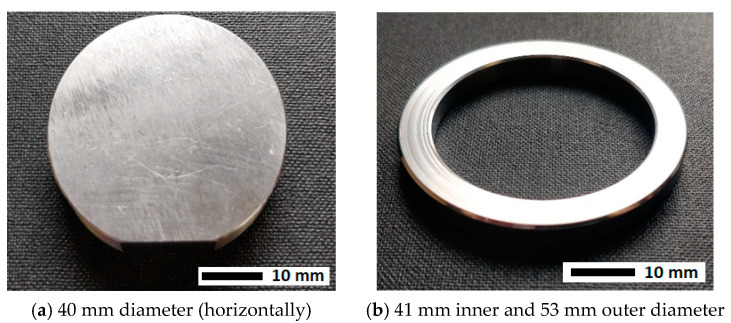
(**a**) Example laboratory test part used for process development; (**b**) Example part for the driveshaft assembly application.

The application uses a ball bearing steel, so laboratory test parts of this material were used for process development, 40 mm diameter and 10 mm thick (see Figure 1a), whilst the ring-shaped parts used in the real-life ship engine application are 53 mm outer diameter, 31 mm inner diameter, and 5 mm thick. The edges of the demonstrator (ring-shaped parts) were chamfered. The surfaces of the samples were ground to a surface roughness of R_a_ 0.4.

Laser texturing was carried using two similar pulsed fibre lasers manufactured by SPI Lasers (now Trumpf), with different average and peak power levels. Initial process development used an SPI redENERGY G4 20 W EP-S laser, with a focused spot diameter of 38 µm (calculated at 1/e2 of maximum intensity), pulse duration of 220 ns, and maximum pulse energy of 0.71 mJ at 28 kHz repetition rate. In the later industrial scale-up stage, the process was adapted for a higher-average-power laser (SPI redENERGY G4 200 W EP-Z). This provided both a larger pulse energy (1.46 mJ) and a higher repetition rate (250 kHz), together with a longer maximum pulse duration of 1020 ns. Hence, with this laser, a significantly higher scanning speed could be used whilst maintaining the same spot-to-spot separation. The focused spot diameter of the 200 W laser is 35 µm (calculated at 1/e2 of maximum intensity).

**Figure 2:**
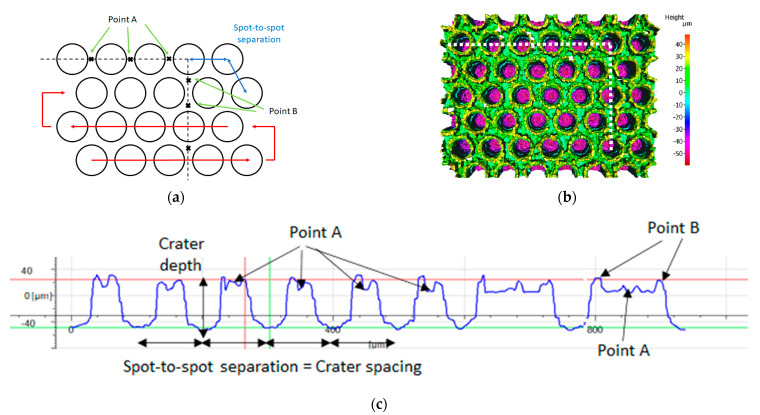
(**a**) Schematic of the laser scanning pattern used for high-friction surfaces. Points A and B are critical when considering surface uniformity. (**b**) Surface profile, with the dotted line indicating measurement of cross-section. (**c**) Cross-section profile of the surface with the key parameters. The first part of the profile is in the x-direction, and the second part of the profile is in the y-direction, as indicated by the dotted line.

The scanning pattern used for laser texturing provides a honeycomb-type surface structure, as shown in Figure 2. To create this, the laser beam was scanned over the sample surface using a galvanometer scan head with an f-theta lens of focal length 160 mm. Various pulse energies, numbers of passes (5 to 30 passes), and spot-to-spot separations were tested (50 to 100 µm). The pulse energies used with the 20 W EP-S laser were 0.36, 0.53, and 0.71 mJ, with a repetition rate of 28 kHz. With the 200 W EP-Z, a pulse energy of 1.46 mJ was used to reduce the number of passes whilst maintaining the required feature depth.

The obtained surface texture is a honeycomb-type pattern of “craters”. Each crater consists of a hole generated by the repeated laser pulses, and melt spatter is deposited at the rim of each hole. The melt spatter forms circular ridges/peaks, which can be pressed into the counterpart to create high friction. In assessing surface texture uniformity, there are two characteristic critical points repeated across the surface, (A) the point between two consecutive craters along the scanning direction and (B) the point equidistant between three craters. These two points should be of a similar height right across the surface to provide the most uniform contact between the surfaces. Moreover, the depth of the features and the spacing between them both have a major influence on the coefficient of static friction, since they directly affect the sharpness of the “peaks” together with the density of features on the surface of the sample.

**Figure 3:**
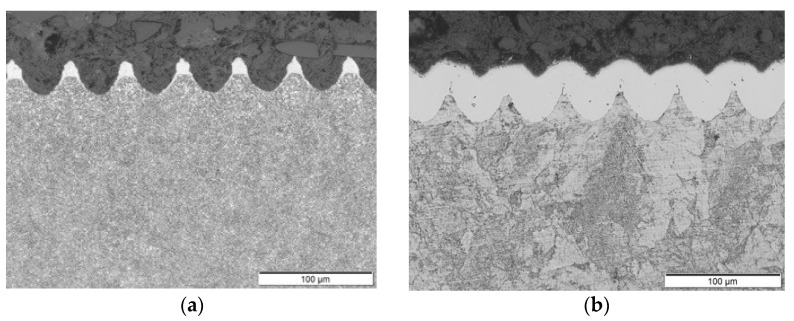
(**a**) LOM cross-section image of the laser-textured sample before hardening. (**b**) The image shows a cross-section of the sample after the TRD CrC hardening process. The sample in the images has a depth of craters ~30 µm and a spot-to-spot spacing ~50 µm.

Surface topographies were measured using an Alicona G3 Surface profilometer. In addition, samples were sectioned through the centre of craters in the scanning direction (see Figure 3), and cross-sectional images were taken using a light optical microscope (LOM).

The hardness of the fully processed, textured, and hardened surface was measured to be in the range 2000–2100 HV 0.025.

**Figure 4:**
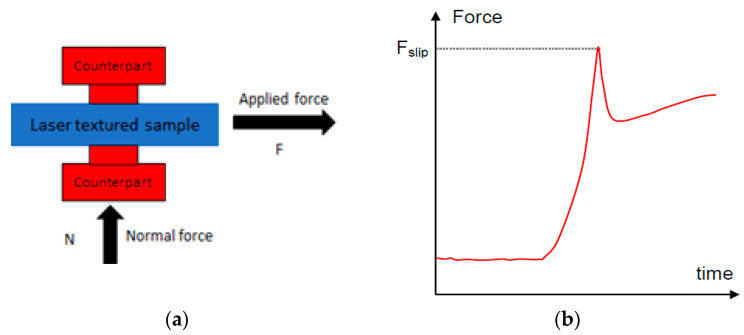
(**a**) Schematic of the static friction coefficient testing setup. (**b**) Typical plot of force as a function of time during the test.

Samples were prepared for static friction testing by laser texturing and TRD hardening on both faces. Friction testing was carried out using the arrangement shown in Figure 4. The processed part was placed in a fixture between two counterpart samples (non-textured), and the normal force N was applied to the fixed counterparts from one side using a load cell (as illustrated). A pulling force F was then applied to the test sample using another load cell, and this was increased until 1mm slip was detected by a motion sensor. The maximum force measured at the point of slippage divided by the normal force and the number of slipping friction surfaces (in this case two) is equal to the coefficient of static friction. The surface contact area was circular with a diameter of 8 mm, and a loading rate of approximately 350 N/s was used.

## 3. Results and Discussion

### 3.1. Impact of Laser Pulse Energy and Amount of Passes

**Figure 5:**
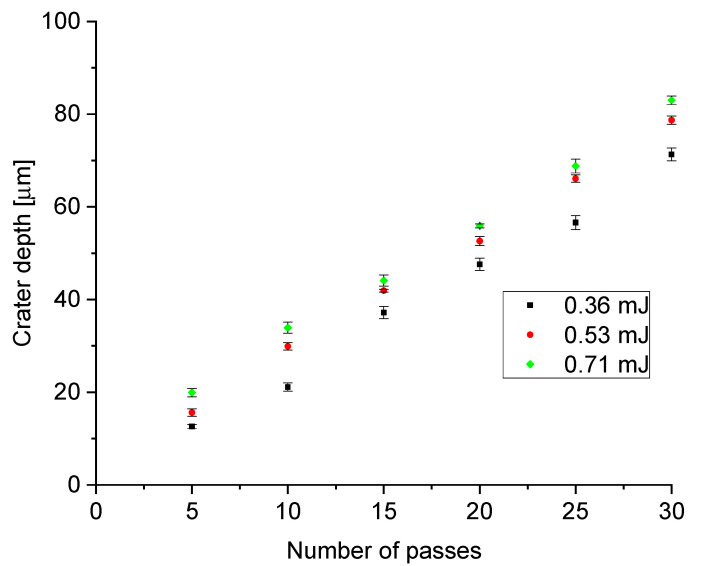
Crater depth as a function of the number of laser passes for three different pulse energies. Results presented for the 20 W laser.

The effects of altering the numbers of laser-scanning passes (5, 10, 15, 20, 25 and 30 passes) and pulse energies (0.36, 0.53 and 0.71 mJ) were studied. A plot of crater depth as a function of the number of laser passes for the three different pulse energies is shown in Figure 5, demonstrating an almost linear trend within the parameter space tested. This indicates that the process can be easily controlled, tailoring the crater depth to provide a particular coefficient of friction suitable for the application.

### 3.2. Impact of Spot-to-Spot Spacing and Surface Uniformity

**Figure 6:**
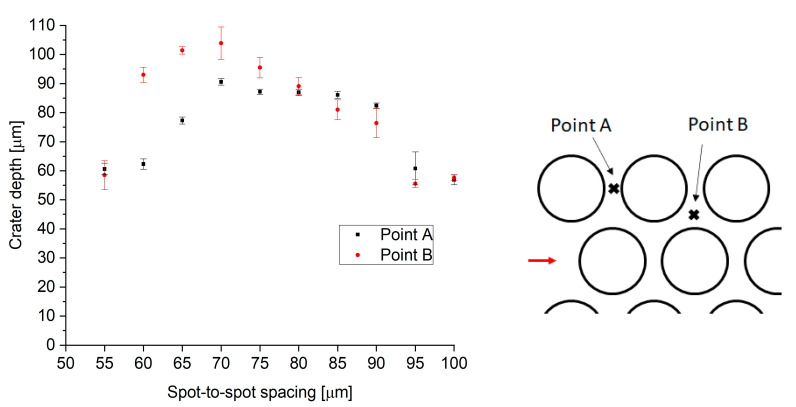
Height of the two key points between adjacent craters. The maximum pulse energy used was 0.71 mJ.

A key requirement for this application is the uniformity of the texture. Precise control over the sample topography together with high uniformity should result in a well-defined and highly reproducible coefficient of static friction. A key parameter to ensure surface uniformity is spot-to-spot spacing, both in the scanning direction and line-to-line. This can be evaluated by measuring the two critical points discussed in Section 2 (see Figure 2). The heights of these points are plotted as a function of spot-to-spot spacing in Figure 6.

**Figure 7:**
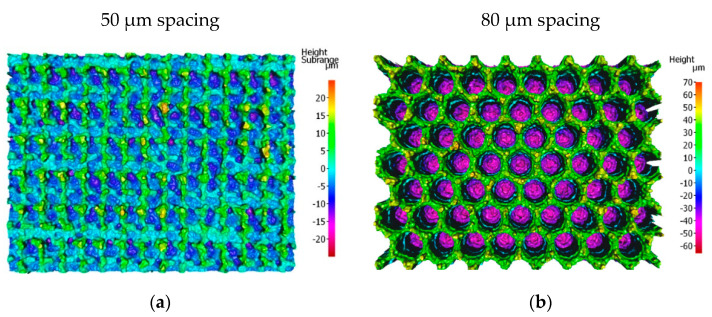
Figure shows the topography of laser structures with (**a**) 50 µm and (**b**) 80 µm spot-to-spot spacing.

For low values of spot-to-spot spacing (50–55 µm), consecutive/adjacent features overlap with the previous ones, leading to features not being fully formed (see Figure 7a). When the spot-to-spot spacing is increased, full well-defined craters are observed (see Figure 7b). As a result, the height of the peaks is also higher for the greater spacings. However, the heights of the peaks at points A and B are significantly different in the intermediate range of spacings from 60 to 75 µm, due to a pile-up of melt at point B. Hence, a spot-to-spot spacing of 80 µm was chosen to provide the most uniform surface, leading to the highest contact area between laser-textured samples and the counterparts.

### 3.3. Route to Industrialisation

**Figure 8:**
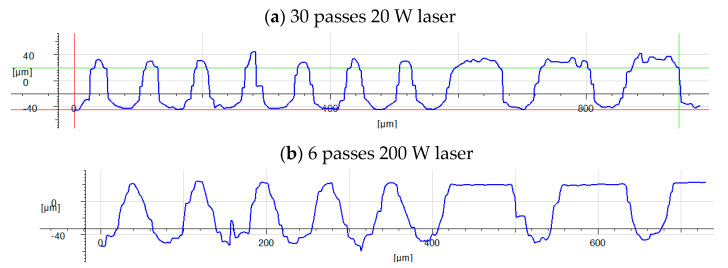
Cross-section surface profile of topographies achieved by the 20 W laser (**a**) and 200 W laser (**b**).

With the 20 W fibre laser, the maximum repetition rate, assuming the maximum pulse energy of 0.71 mJ at the 220 ns pulse duration, was limited to 28 kHz. To achieve a satisfactory surface structure on both sides of the high-friction ring, a total of 60 passes are required with these parameters (30 passes on each side). Using these parameters, two fully laser-textured parts could be produced in one hour. To increase the processing rate (up to 15 fully textured parts in an hour) and hence reduce costs, it is necessary to use the 200 W laser with a pulse energy of 1.46 mJ, a repetition rate of 70 kHz, and a scan speed of 5.4 m/s. The goal was to maintain the surface topography (as shown in Figure 7a) while increasing processing speed. Figure 8 shows the surface cross-section profile for structures generated by both 20 W and 200 W lasers.

**Figure 9:**
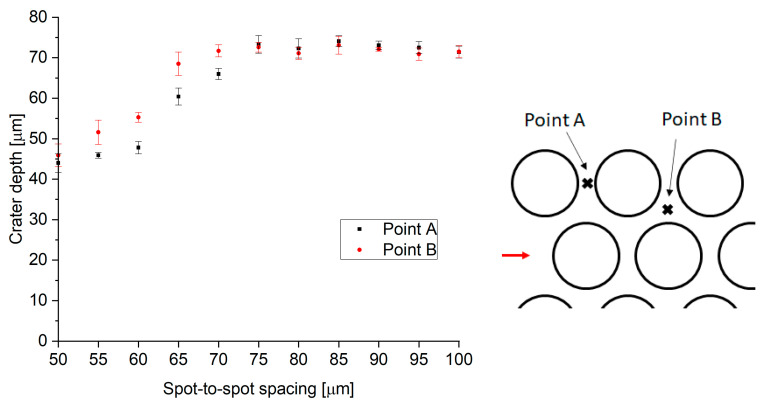
Height of the two key points between adjacent craters. The pulse energy used was 1.46 mJ, with the 200 W SPI laser.

This change of laser source alters the processing regime. With the 20 W laser, melt ejection from the crater creates the top surface topography around the crater, whereas the 200 W laser produces a cleaner crater with much less recast material around the crater because the melt is expelled with greater velocity and so does not form significant recast around the edges of the crater. This results in a more uniform surface, whilst the higher pulse energy also creates deeper features. The crater depth is plotted as a function of spot-to-spot spacing in Figure 9.

**Figure 10:**
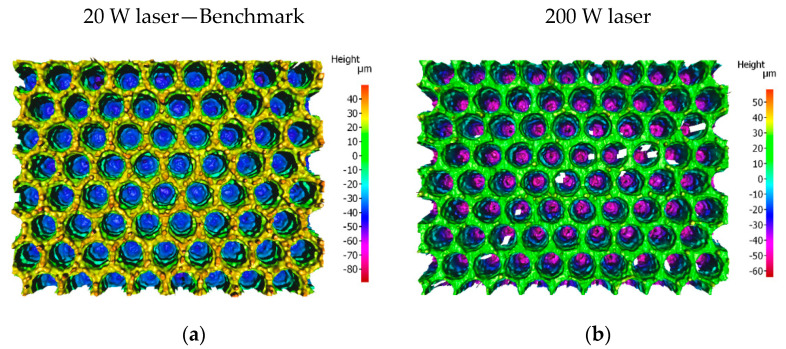
Comparison of the surface topographies for processing with 20 W laser (**a**) and 200 W laser (**b**).

For a spot-to-spot spacing of at least 75 µm, the height of both critical points is almost the same to within a few microns. With almost no recast around the edges of the craters, these peaks are essentially the original surface of the sample. By eliminating the randomness associated with melt dynamics and recast structures, a much flatter top surface of the sample is achieved, thus minimising the variance of structures produced. Figure 10 shows surface profiles of the surface textured with the 20 W and 200 W lasers for comparison.

### 3.4. Coefficient of Static Friction

**Figure 11:**
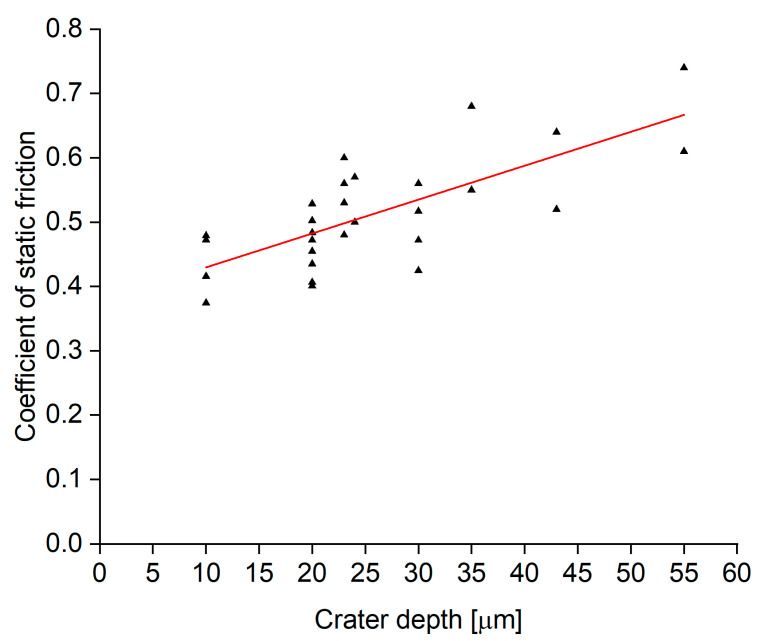
Static friction coefficient as a function of crater depth. The results presented here were obtained with both the 20 W and 200 W lasers. Results include various spot-to-spot spacings. The results for the same crater depth are for a range of different spot-to-spot spacings, hence the spread of coefficient of friction values.

**Figure 12:**
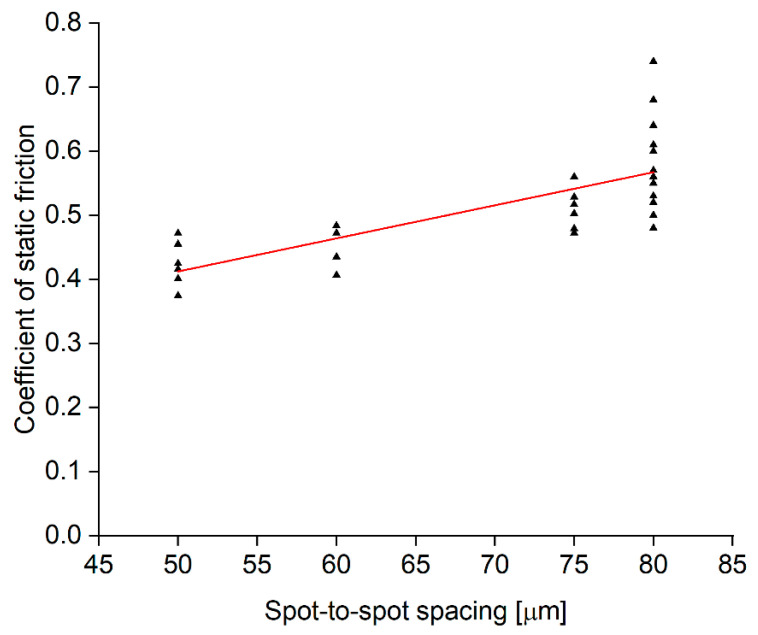
Static friction coefficient as a function of spot-to-spot spacing. The results presented here were obtained with both the 20 W and 200 W lasers. The results for the same spot-to-spot spacing are for a range of different crater depths, hence the spread of coefficient of friction values.

The requirement for the high-friction rings is to have a coefficient of static friction of at least 0.60 to fulfil their function within the driveshaft assembly. Static friction measurements were made across the full range of laser parameters tested, and these are grouped in the plots shown in Figure 11 and Figure 12, where the coefficient of friction is presented as a function of the pattern height and spot-to-spot spacing, respectively. The results shown in Figure 11 indicate a clear linear trend, in that an increased peak height leads to an increased coefficient of static friction.

The results presented in Section 3.2 indicate that a spot-to-spot spacing of around 80 µm would create the most uniform surface and, as a result, provide the most uniform contact between the surface of the sample and the counterpart. This is confirmed when looking at the trend in Figure 12. For multiple crater depths, the best results were obtained for a spot-to-spot spacing of around 80 µm, where the texture has the most uniform (height of peaks in point A and point B is most similar) top surface. Figure 12 shows the coefficient of static friction as a function of spot-to-spot spacing for both 20 W and 200 W lasers.

A fundamental process parameter *P_text_* can be defined as a combination of the three key parameters:

(1) $$P_{text}=\frac{spot-to-spot\;spacing\times crater\;depth}{chromium\;carbide\;layer\;thickness}$$

**Figure 13:**
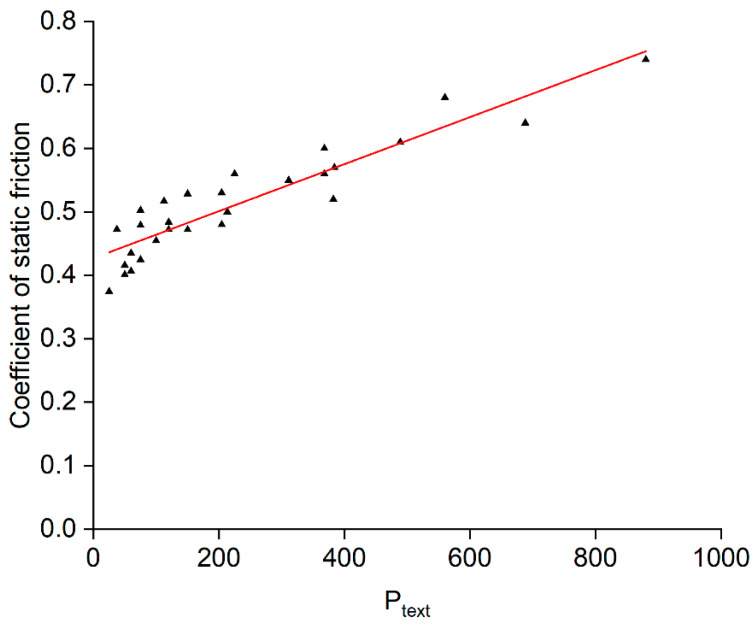
The processing window when using both 20 W and 200 W lasers.

This fundamental parameter enables the process to be transferred between laser equipment of differing pulse parameters, as shown in the graph plotted in Figure 13, in which the coefficient of static friction is plotted as a function of *P_text_* for three different thicknesses of the hardening layer. This allows the selection of suitable parameters for a given laser source in order to obtain a particular coefficient of friction value.

### 3.5. Reproducibility

For this process to be industrially relevant for ship engine application, it must provide an increased reproducibility of the static friction coefficient. The currently used thermal spray process has achieved a relative standard deviation of 13–14%, whereas for the laser textured samples (with hardening), a relative standard deviation was measured at 3–4% for a single set of parameters. This is a significant reduction in variation—an increase in reproducibility which exceeds the expectations.

### 3.6. Demonstrator Testing

Test rings were textured with our developed process and directly compared with thermally sprayed parts. The thermal-sprayed discs had a standard WC-Co-Cr coating applied by a High Velocity Oxygen Fuel (HVOF) process. To investigate the slippage process, an increasing torque was applied to the driveshaft unit (with the high-friction ring inside) until the part slipped. The thermal-sprayed discs were tested using the same conditions as the laser-textured discs. The surfaces of the slipped counterparts were subsequently analysed. The parameters that we have used for laser texturing of final components are as follows: 80 µm spot-to-spot spacing, 1020 ns pulse duration, 1.46 mJ pulse energy, and 70 kH processing rate.

**Figure 14:**
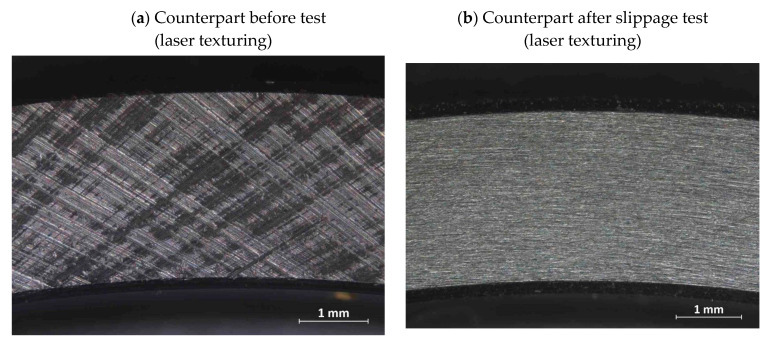

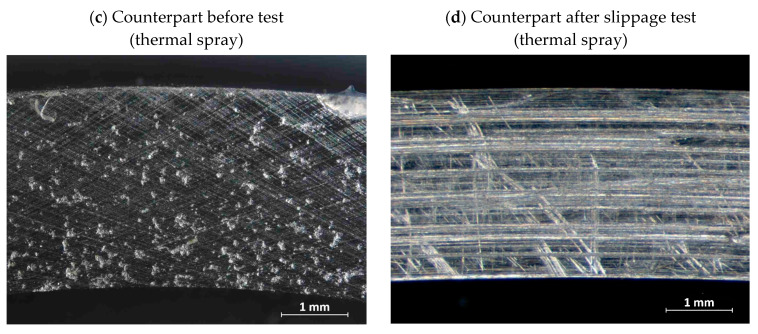
Images of counterparts that are in contact with the high-friction rings before and after slippage test. (**a**) Counterpart before slippage test (laser texturing)—no visible indentations by pressing of the laser textured part. (**b**) Counterpart after slippage test (laser texturing)—The surface of the counterpart after the slip test with laser- textured and hardened part exhibits a uniform surface without any dominant features. (**c**) Counterpart before slippage test (thermal spray)—The bright spots are indentations created by pressing of the thermal-sprayed part into the counterpart, indicating uneven, large structures created by the coating. (**d**) The surface of the counterpart after the sliptest with the thermal-sprayed sample exhibits large scuff marks and scratches created by the thermal-sprayed surface.

Figure 14 shows images of the counterpart surfaces before and after the test for both laser-textured and thermal-sprayed parts. These images provide a good indication of the uniformity of the high-friction ring surface. The surface of the counterpart that was in contact with the laser-textured/hardened parts has a uniform surface without any major streaks or marks. The counterpart that was in contact with the thermally sprayed parts has many highly visible scuff marks. This is an indication that the thermally sprayed surface is significantly more uneven, with a few larger-scale structures, which is likely to result in greater variability in the coefficient of static friction (as reflected in the significantly higher standard deviation).

### 3.7. Final Ship Engine Testing at Sea and Plans for Commercialisation

After successful laboratory testing, a service test was carried out, using the textured rings in ship trials. The rings were mounted in 2 × 3 driveshafts in two different marine engines on ships. The requirement for parts acceptance was set at “1500+” hours of operation. The parts performed without any problems, and the driveshafts were subsequently examined. This examination confirmed that the parts had undergone no slippage and therefore performed correctly. Consequently, the process is considered a suitable replacement for the currently used process, as it offers improved uniformity and reproducibility. These ship tests formed the final approval step for this novel two-step texturing and hardening process, and it is planned to use this process as the new standard in driveshaft application. Moreover, due to the improved functionality of the friction ring, a set of new, more stringent specifications has been defined for this application. The previous requirement was simply that the coefficient of static friction be at least 0.6, whereas it is planned that the new specification will require the coefficient of static friction to be within a range of 0.7 to 0.9.

## 4. Conclusions

We have successfully developed a practical two-step process (laser surface structuring and subsequent deposition of a hard chromium carbide layer) for the manufacture of well-defined high-friction surfaces, with a focus on an application in driveshafts of marine engines. Process development focused on providing improved reproducibility compared with the current solution of thermal spraying. A laser surface-texturing process is used to provide a suitable surface topography, and results are presented for two different nanosecond-pulsed laser systems, 20 W and 200 W. It is possible to successfully create high-friction surfaces with either laser, but the 200 W laser significantly decreases the laser processing time, making it more industrially viable. We have achieved a coefficient of static friction of 0.74 (0.6 requirement) and a relative standard deviation of 3–4% as opposed to 13–14% for thermally sprayed samples.

Following the laboratory and demonstrator slippage tests, the parts were installed in driveshafts for ship testing. After successfully performing for over 1500 h, and further examination of the surface, laser texturing plus chromium carbide deposition has been qualified as the new standard manufacturing process for high-friction rings for these marine driveshaft assemblies.

## Data Availability

Data available in a publicly accessible repository (Once approved) Heriot-Watt Research Portal https://researchportal.hw.ac.uk/en/.
